# Evaluation of Imaging Software Accuracy for 3-Dimensional Analysis of the Mandibular Condyle. A Comparative Study Using a Surface-to-Surface Matching Technique

**DOI:** 10.3390/ijerph17134789

**Published:** 2020-07-03

**Authors:** Antonino Lo Giudice, Vincenzo Quinzi, Vincenzo Ronsivalle, Marco Farronato, Carmelo Nicotra, Francesco Indelicato, Gaetano Isola

**Affiliations:** 1 Department of General Surgery and Surgical-Medical Specialties, Section of Orthodontics, School of Dentistry, University of Catania, 95123 Catania, Italy; nino.logiudice@gmail.com (A.L.G.); vincenzo.ronsivalle@hotmail.it (V.R.); gnicotra@unict.it (C.N.); 2 Post Graduate School of Orthodontics, Department of Life, Health and Environmental Sciences, University of L’Aquila, V.le San Salvatore, 67100 L’Aquila, Italy; vincenzo.quinzi@univaq.it; 3 Department of Medicine, Surgery and Dentistry, Section of Orthodontics, University of Milan, 20122 Milan, Italy; marcofarronato@msn.com; 4 Department of General Surgery and Surgical-Medical Specialties, Section of Oral Surgery and Periodontology, School of Dentistry, University of Catania, 95123 Catania, Italy; indelicato@policlinico.unict.it

**Keywords:** 3D rendering, condyle, segmentation, 3D printing, cone-beam computed tomography, threshold, field of view

## Abstract

The aim of this study was to assess the accuracy of 3D rendering of the mandibular condylar region obtained from different semi-automatic segmentation methodology. A total of 10 Cone beam computed tomography (CBCT) were selected to perform semi-automatic segmentation of the condyles by using three free-source software (Invesalius, version 3.0.0, Centro de Tecnologia da Informação Renato Archer, Campinas, SP, Brazil; ITK-Snap, version2.2.0; Slicer 3D, version 4.10.2) and one commercially available software Dolphin 3D (Dolphin Imaging, version 11.0, Chatsworth, CA, USA). The same models were also manually segmented (Mimics, version 17.01, Materialise, Leuven, Belgium) and set as ground truth. The accuracy of semi-automatic segmentation was evaluated by (1) comparing the volume of each semi-automatic 3D rendered condylar model with that obtained with manual segmentation, (2) deviation analysis of each 3D rendered mandibular models with those obtained from manual segmentation. No significant differences were found in the volumetric dimensions of the condylar models among the tested software (*p* > 0.05). However, the color-coded map showed underestimation of the condylar models obtained with ITK-Snap and Slicer 3D, and overestimation with Dolphin 3D and Invesalius. Excellent reliability was found for both intra-observer and inter-observer readings. Despite the excellent reliability, the present findings suggest that data of condylar morphology obtained with semi-automatic segmentation should be taken with caution when an accurate definition of condylar boundaries is required.

## 1. Introduction

Morphological and dimensional changes of the mandibular condyles can alter craniofacial growth, sometimes favoring the development of skeletal malocclusions, or can be a specific risk factor for temporomandibular disorders (TMD) [1,2,3]. Orthognathic surgery can also induce changes in this area in the form of both physiological and pathological responses [4,5]. Conversely, changes in the condylar area are sometimes desired, for example, during functional orthopedic treatments planned for stimulating, inhibiting, or modifying mandible growth pattern [1,6,7,8]. All these clinical circumstances required an accurate evaluation of condylar morphology and dimension to obtain useful information for a comprehensive diagnosis or to assess treatment plan effectiveness [3,6,9,10].

Magnetic resonance imaging (MRI) and computed tomography (CT) represent the gold standard imaging modality for evaluating temporomandibular joint (TMJ) dysfunctions respectively related to soft-tissue ore bony imbalance [11,12]. However, MRI presents intrinsic limitations in the assessment of osseous modifications of TMJ [11] while CT involves high radiation dose and relatively limited availability in private practice settings [12]. In this respect, cone-beam computed tomography (CBCT) represents the 3D imaging method of choice in oral and maxillofacial fields [12,13], due to its high accuracy in detecting bone characteristics at lower risk of radiation exposure [14,15].

Different methods have been described for segmentation of the mandibular condyles, basically, they can be distinguished in manual and semi-automatic procedures [15,16,17,18,19,20]. In the manual segmentation, the operator outlines the condylar contour slice by slice, distinguishing the condyle from adjacent tissues [1,16]. In general, this procedure is time-consuming and has shown great accuracy when managed by expert operators [1]. Conversely, the semiautomatic segmentation is a computer-aided approach (hybrid) where the operator preliminary selects the threshold interval (Hounsfield units) that guides the automatic 3D rendering procedure [21]. Semi-automatic segmentation can be performed by using the binary threshold-based volume [18,19] or the region-growing algorithm [15,17]. In the first method, the operator uses specific tools to remove structures outside the volume of interest, while in the second method the user places specific seed-points through different slices that start “growing” or “expand” based on the preliminary threshold set. However, literature is still lacking uniform data confirming the accuracy of semiautomatic segmentation of the mandibular condyles [1]. The main reason behind this inconsistency is related to the different methodologies involved [1] as well as the difficulty in the segmentation process of a complex region such as the mandibular condyles [18,19].

In the absence of real anatomic structure (dry mandible) or its realistic reproduction (laser scanning), manual segmentation represents the gold standard for 3-dimensional reconstruction. In fact, it allows the detection of areas with low bone density or with not well-defined boundaries due to low-contrast and proximity with other structures [18]. However, semiautomatic segmentation is faster than a manual approach, which is relevant from a clinical perspective. Additionally, semi-automatic segmentation is not influenced by intra-operator reliability [22], which is also important for clinical and research purposes. In this respect, the aim of the present study was to assess the accuracy of four software (three free sources and one licensed) for semi-automatic segmentation of the mandibular condyle, in comparison with a manual segmentation approach. For this purpose, we referred to a specific 3D digital diagnostic technology involving the surface-to-surface matching and deviation analysis [10,23,24,25] of 3D rendered condylar models. The null hypothesis was the absence of significant differences in the accuracy of semi-automatic segmentation software compared to manual segmentation.

## 2. Materials and Methods

This study followed the Helsinki Declaration on medical protocols and ethics and received positive response by the Approval Board of the School of Dentistry, University of Catania (protocol n. 14/19). The study sample included 10 subjects (six females, four males; mean age 26.4 ± 3.9 years old) who were referred for surgically assisted rapid maxillary expansion treatment; therefore, patients were not unnecessarily subjected to additional radiation. Inclusion criteria were as follows: subjects between 18 and 40 years old, good quality CBCT scans, a field of view (FOV) including the whole mandibular bone. Exclusion criteria: images artefacts or images distortion, craniofacial deformities, mandibular functional shift signs or symptoms of the temporomandibular joint disorder, third molar impaction and dental implants, previous orthodontic treatment. Patients were scanned with the same iCAT CBCT Unit (Imaging Sciences International, Hartfield, PA). The setting protocol included 0.3 voxel, 8.9 s, large field of view at 120 kV and 20 mA. The distance between two slices was 0.3 mm, which provided accuracy in anatomic registration.

After head orientation [22], five CBCT viewer software programs were used to segment and generate 3D rendering of patients’ mandibular bone. A preliminary segmentation of the mandible was necessary for accurate and reproducible registration of the condylar region for surface deviation analysis (see the description of the digital work-flow). The segmentation of the mandible was performed by selecting the adequate threshold interval for visualizing this anatomical region (semi-automatic). Then, a second mask limited to the condylar region (ROI set below the sigmoid notch) was generated according to the different methodologies tested in this study (manual and semi-automatic) and was merged with the mask obtained from the segmentation of the mandible. Each software tested in the present study allowed to perform this procedure, except for the Dolphin software; in this case, a single mask was generated using the best threshold range for detecting the condylar region as reference for the segmentation of the total mandible.

**Figure 1:**
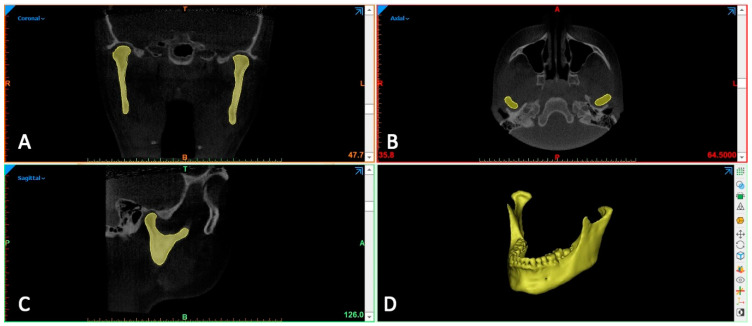
Manual segmentation of mandibular jaw (ground truth); (**A**) coronal view; (**B**) axial view; (**C**) sagittal view; (**D**) 3D rendered mandible model.

In particular, the Mimics software (version 17.01; Materialise, Leuven, Belgium) was used to perform a fully manual segmentation of the condyles which served as ground truth (gold standard) of the present investigation (Figure 1). Semi-automatic segmentation of the mandible was carried out by using four software, i.e., Dolphin3D (Dolphin Imaging, version 11.0, Chatsworth, CA, USA), Invesalius (version 3.0.0; Centro de Tecnologia da Informação Renato Archer, Campinas, SP, Brazil), ITK-Snap (version2.2.0; www.itksnap.org), and Slicer 3D (http://www.slicer.org). Segmentations were performed according to each software manufacturer’s recommendations and using the interactive threshold technique which means that operator selected the best threshold interval for visualizing the entirety of the anatomic boundaries of the condyles. In particular, segmentation was performed with the binary threshold-based algorithm (Dolphin 3D and Invesalius) and with the region growing algorithm (ITK-Snap and Slicer 3D). Once the segmentation mask was obtained in each software, it was rendered into a 3D model and exported as STL ASCII electronic format. Description of each software program is shown in Table 1.

To determinate the accuracy of the semi-automatic segmentation performed with each software, a surface deviation analysis was conducted by superimposing each obtained condylar shell with the ground truth condylar shell (obtained from manual segmentation) [21,22,23]. Briefly, the workflow of the 3D deviation analysis is explained in the following steps.

### 2.1. Step 1—3D Model Superimposition and Final Registration

**Figure 2:**
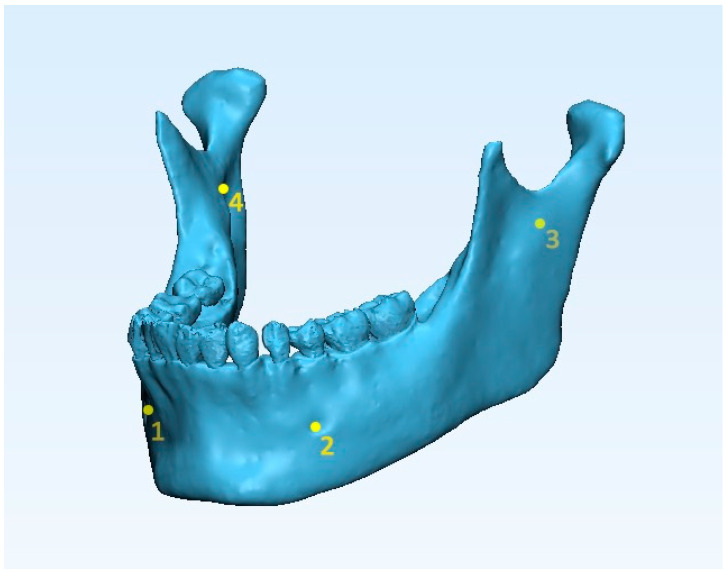
Landmarking four points on 3D mandibular model superimposition: 1–2, the geometric center of Table 3. left and right mandibular lingual at the inner surface of ramus.

The mandibular 3D models were imported onto 3-matic research software (vr. 11.0.0.109, Materialise NV, Liege, Belgium). Each mandibular model obtained from tested software (semi-automatic segmentations) was superimposed to the ground truth mandibular model (manual segmentation). A preliminary registration was carried out by selecting the same four points on the surface of the 3D models (Figure 2): (1–2) the geometric center of the left and right metal foramina; (3–4) left and right mandibular lingual at the inner surface of ramus. Then, to enhance the quality of the superimposition, a surface-based registration was made by using the ‘Best fit alignment’ function. Using the ground truth mandibular model as the reference, the final superimposition was carried out by setting the precision as at least 0.01 mm.

### 2.2. Step 2—Definition of 3D Model of the Condyle (Exclusion of the Mandible and Coronoid Process)

**Figure 3:**
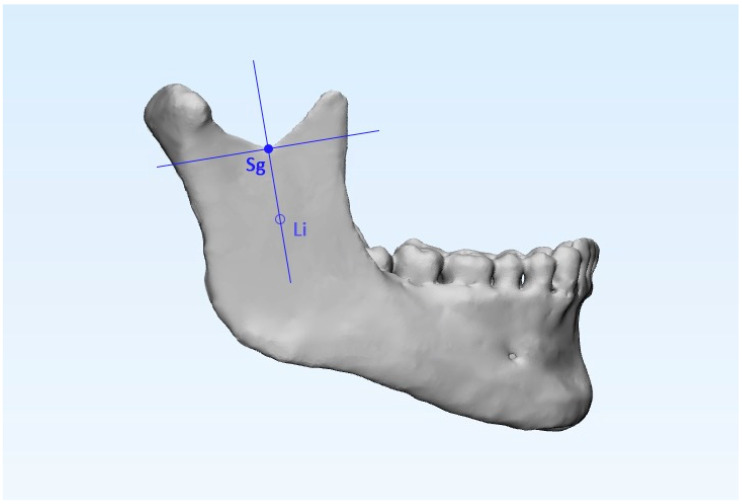
The sigmoid point (Sg) and the point representing the external projection of the lingual (Li) were used to generate two plane cuts, respectively, the Sg-Li plane and a perpendicular plane passing through Sg (90° with respect to the Sg-Li). These two planes were used to delineate the condylar region.

Specific landmarks were identified on the reconstructed 3D image in order to enable delimitation of each condylar unit, in particular the sigmoid point (Sg) and the external projection of the lingual (Li). To delineate the condylar region (Co), a perpendicular line (90°) to Sg-Li passing through Sg was drawn (Figure 3) [26].

### 2.3. Step 3—Final Registration, Surface-Based.

**Figure 4:**
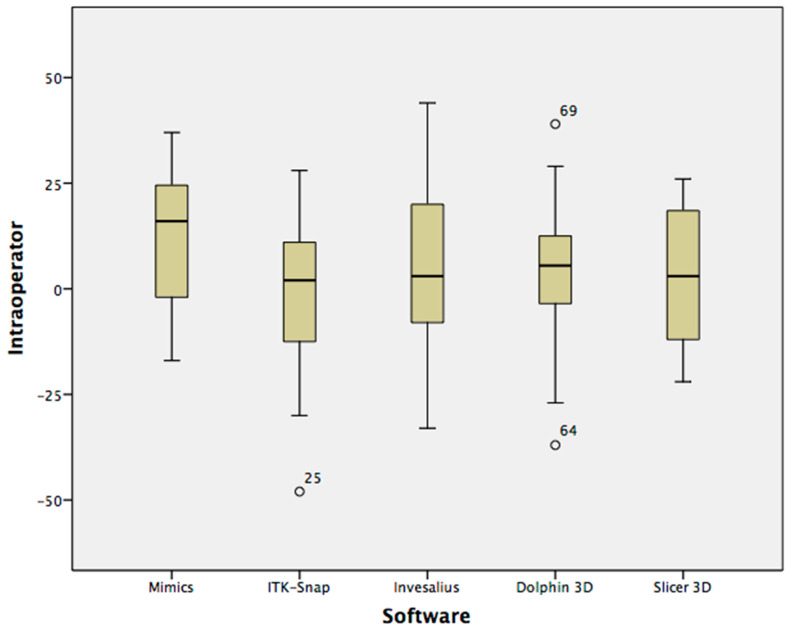
Box-Whisker plots of volumetric differences between first and second intra-operator readings.

**Figure 5:**
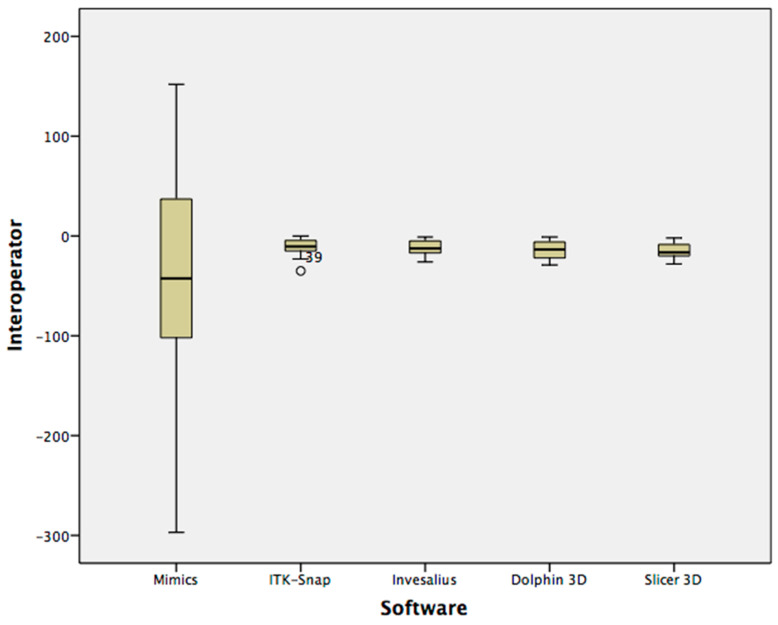
Box-Whisker plots of volumetric differences between first and second inter-operator readings.

Subsequently, surface-based deviation analysis was carried out using the specific function in the Geomagic Control X software (version 2017.0.0, 3D Systems, Santa Clara, CA 95054, USA). The analysis was complemented by visualization of the 3D color-coded maps set at two range of tolerance, respectively, 0.3 and 0.6 mm, to better evaluate and locate the discrepancy between the model surfaces (Figure 4 and Figure 5). The maximum deviation calculation was set to 1.00 mm. After the deviation analysis, percentages of all the distance values within the tolerance range were calculated. The software also allowed the calculation of total volume of the 3D models of the mandibular bone. These data were recorded on a spreadsheet and used for comparative analyses.

### 2.4. Step 4—Matching Percentage Calculation

Once the deviation analysis was carried out, the percentages (%) of all the distance values were calculated for the two tolerance groups. These values represented the degree of matching between the two models and, thus, shows the accuracy of mandibular models obtained with tested software (semi-automatic segmentation).

The work-flow segmentation and relative mask generation were carried out by two operators, respectively, with 15 years (A.L.G.) and 3 years (V.R.) of experience in 3D imaging in cranio-facial field, in order to assess inter-operator variability according to the degree of expertise. The images were re-measured 4 weeks after completing the first measurements in order to assess intra-operator reliability and separate spreadsheets were generated in order to blind the operator from previous data.

### 2.5. Statistical Analysis

A preliminary evaluation of sample size power was performed on 10 condyles (five CBCT examination), the analysis suggested that 16 condyles were required to reach the 80% power to detect a mean difference of 21.07 mm^3^ in the volumetric assessment of the condylar region between manual segmentation and semi-automatic segmentation (ITK-Snap software), with a confidence level of 95% and a beta error level of 20%. However, according to the inclusion criteria, we were able to include 20 condyles (10 CBCT examinations) which increased the robustness of the data.

The normal distribution and equality of variance of the data was performed with Shapiro–Wilk Normality Test and Levene’s test. Since data were not normally distributed neither showed equality of variance and non-parametric statistical tests were used. In particular, the Kruskal–Wallis test was used to assess (1) differences in the volumetric dimension of the 3D rendered condylar models obtained with each software investigated, (2) the differences in the matching percentage between the mandibular models obtained with semi-automatic software and the ground truth mandibular model (manual segmentation). Mann–Whitney U test was used to perform post-hoc comparison tests. Wilcoxon Signed-Rank Test was used to assess intra-operator and inter-operator differences in the volumetric readings of the 3D rendered condylar models. Finally, intraclass correlation coefficient (ICC) was used to check the reliability of the first and second measurements. Data were analyzed using SPSS^®^ version 24 Statistics software (IBM Corporation, 1 New Orchard Road, Armonk, NY, USA) with a significance level set at *p* < 0.05.

## 3. Results

No statistically significant differences were found in the volumetric dimensions of the rendered condylar models obtained with different software, with manual segmentation (Mimics/ground truth model) showing a median value of 1631 mm^3^ and semiautomatic segmentation showing median values ranging from 1602 (ITK-snap) to 1662 mm^3^ (Invesalius) (Table 2).

Significant differences were found in the percentage of matching of the condylar models obtained with different semi-automatic software relative to the ground truth condylar models, at both ranges of tolerance (Tolerance A = 0.3 mm, *p* < 0.001; Tolerance B = 0.6 mm, *p* < 0.05) (Table 3). The matching percentage ranged from 61% (Slicer 3D) to 69% (ITK-Snap) at a range of Tolerance A and from 78.50% (Dolphin3D) to 84.77% (ITK-Snap) at a range of Tolerance B. 

Concerning intra-operator differences, only manual segmentation showed a statistical significance between first reading (1631 mm^3^, median value) and second reading (1624 mm^3^, median value) (*p* < 0.05) (Table 4).

Concerning inter-operator differences, only manual segmentation showed a statistical significance between the first reading (1631 mm^3^, median value) and the second reading (1700 mm^3^, median value) (*p* < 0.001) (Table 5).

Figure 4 and Figure 5 show the volumetric differences (Box-Whisker plots) between the first and second readings with each software tested, respectively, for intra-operator and inter-operator assessments.

The ICC values showed excellent agreement for intra-observer reliability, ranging from 0.993 to 0.997 among all the tested software (Table 6). For inter-observer reliability, the ICC values showed good agreement of the manual segmentation (0.856) and excellent agreement for semi-automatic segmentation, ranging from 0.996 to 0.998 (Table 6).

**Figure 6:**
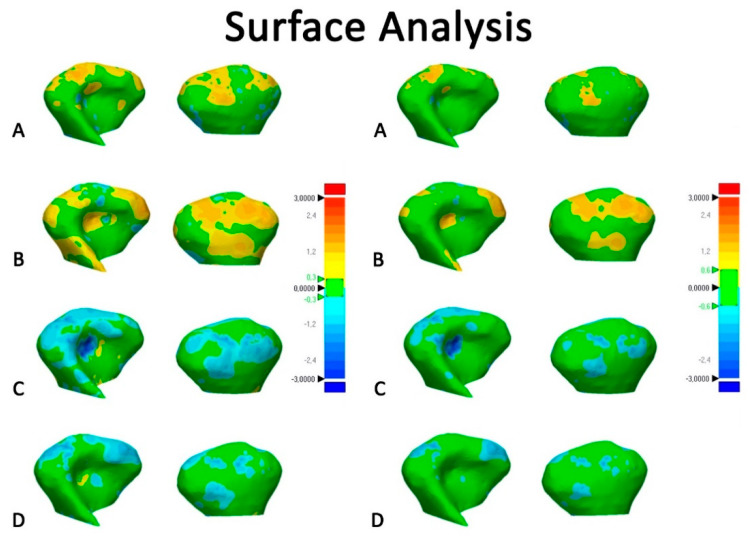
Surface-based deviation analysis between 3D condylar models obtained with semi-automatic segmentation and its ground truth model (manual segmentation). (**A**), Invesalius; (**B**), Dolphin 3D; (**C**), ITK-Snap; (**D**), Slicer 3D. The green of the color scale bars represents the range of tolerance. Left side: Color map set to a range of tolerance of ±0.3 mm. Right side: color map set to a range of tolerance of ±0.6 mm.

**Figure 7:**
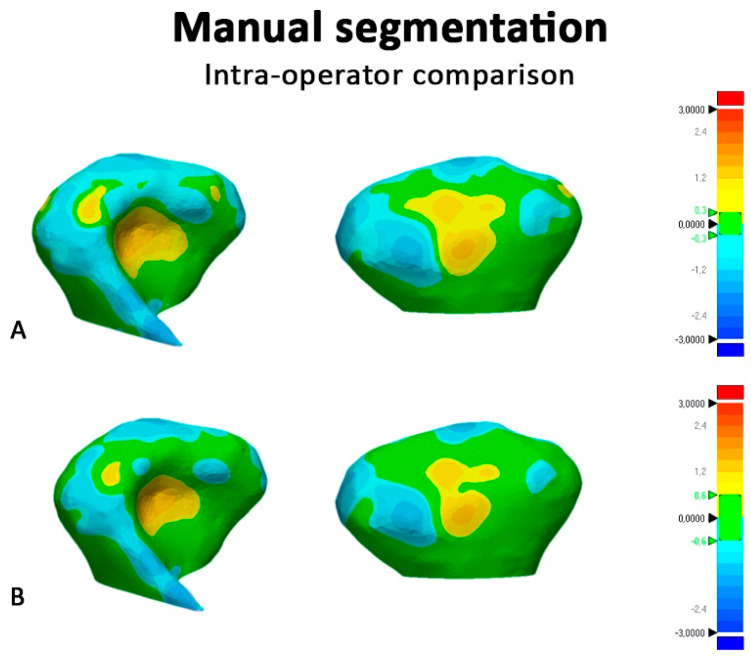
Surface-based deviation analysis between 3D condylar models obtained with manual segmentation performed respectively by two operators with different levels of expertise. The green of the color scale bars represents the range of tolerance. (**A**), Color map set to a range of tolerance of ±0.3 mm Right side. (**B**), Color map set to a range of tolerance of ±0.6 mm.

Figure 6 and Figure 7 show the color-coded maps obtained, respectively, from (1) surface-based deviation analysis between semi-automatic segmentation and manual segmentation, (2) surface-based deviation analysis between manual segmentations performed by two operators with different levels of expertise.

## 4. Discussion

To date, the market proposes a plethora of software for analyzing digital imaging communications in medicine (DICOM) from CBCT scans, most of them including semi-automatic segmentation tools. Free-source DICOM viewers are also available online. Most of them included specific segmentation algorithm and were developed in a university setting or in a small research group, as a consequence, dental clinicians may not be aware of such open-source options. In the present study, three free-source software (Invesalius, ITK-Snap, Slicer 3D) were tested along with one commercially available software (Dolphin3D) specifically developed for orthodontic/maxillofacial diagnostic purposes.

In particular, we decided to investigate the accuracy of semi-automatic segmentation of the mandibular condyles due to the documented difficulty in defining this anatomical region for 3D rendering. This has been attributed to the low density of the bone in the condylar area, the overlapping cranial base bony structures, and proximity to the articular disc [1,18,19]. Moreover, the cone-beam geometry hampers the spatial resolution of the condylar region due to higher noise in peripheral areas of the CBCT scan [27] and manual adjustment is often required for complete contouring in the absence of full manual segmentation process [15,20,28]. In fact, to assess the accuracy of semi-automatic segmentation of the condyles, we referred to the manual segmentation as ground truth model, which represents the gold standard for 3D rending in the absence of real anatomic structure (dry condyles) or its realistic reproduction (laser scanning) [1,19].

According to the present findings, no significant differences were found in the volumetric reconstruction of the condyles between semi-automatic and manual segmentation, these differences being in a small range between - 31 mm^3^ and + 24,60 mm^3^ (as median values). In this regard, Dolphin 3D and 3D Slicer software show a slight underestimation and ITK-Snap and Invesalius software show a slight overestimation of volumetric data. However, volumetric data do not provide a qualitative evaluation of the accuracy of anatomical CBCT-derived models, i.e., precise information of the area of discordance between two surface models. This information is relevant when assessing the morphology of the condyles, especially if we consider that the main difficulty during segmentation of this region is related to the ability in distinguishing the contour from the articular disc and/or bony structures [1,20,29,30,31]. Thus, the accuracy of semi-automatic segmentation was also assessed by the superimposition of the 3D models obtained from each investigated software with those acquired with manual segmentation. Afterwards, deviation analysis was used to detect shape differences between the two CBCT-derived condyles models as well as to obtain precise dimensional information, according to a consolidated methodology [10,23,26].

3D rendered mandibular condyles obtained from semi-automatic segmentation showed moderate matching with respect to manual segmentation, in a range between 61% and 69%, according to the range of tolerance of 0.3 mm (Tolerance A). The color-coded map showed areas of underestimation (turquoise-to-dark blue) of the condylar surface obtained with ITK-Snap and Slicer 3D, conversely, it showed the area of overestimation of the condylar surface obtained with Dolphin 3D and Invesalius (Figure 6, left side), confirming the data obtained from the volumetric assessment. The percentage of matching increased when deviation analysis was set at 0.6 mm (Tolerance B), the green area being more extended with this range of tolerance (Figure 6, right side). This means that the surface error of 3D semi-automatic rendered condyles was barely around the clinically accepted error margin of 0.5 mm [1,32]. As a consequence, there was no safe interval with respect to the clinically accepted error to fully trust the automatic segmentation for an accurate evaluation of condylar morphology. In this respect, such differences could alter the clinical responsiveness, for example, in the diagnosis of condylar hyperplasia, condylar arthritis, and asymmetry of the ramus of the mandible [9,23,33,34,35,36,37,38].

In this study, the main factors potentially causing differences between condylar models obtained with semi-automatic and manual segmentation were the threshold selection methods as a systematic error and operator variability as random error. In this respect, the image scans included in the study were obtained from the same CBCT machine, with the same acquisition parameters. Thus, all factors affecting the accuracy of 3D model rendering prior to segmentation process were controlled and limited to the usage of different software [13,22,39].

The semi-automatic segmentation process is dependent on spatial and contrast resolution of the scan, the thickness and degree of calcification or cortication of the bony structure and the software algorithm [35]. Bearing this in mind and considering the present findings, it is possible that software based on growing region algorithm (Slicer 3D, ITk-snap) could present difficulties in completing an accurate detection of the condylar boundaries since threshold-based seed-points do not cover hypodense voxels in this region. On the contrary, software based on threshold-based segmentation algorithm (Dolphin 3D and Invesalius) could cause an overestimation of condylar boundaries since the segmentation procedure still relies on the operator’s visual discrimination of the bony structure and definition of threshold-level [1,40,41].

Concerning the operator-dependent error, semi-automatic segmentation showed excellent intra-operator and inter-operator reliability according to the ICC values and to the small volumetric differences found between the first and the second recordings. In this respect, the semi-automatic segmentation delegated most of this task to the software algorithm [1,17], this reduced the magnitude of the observer related error. For the same reason, semi-automatic segmentation almost voided the difference between the readings performed by two observers with different level of expertise in 3D imaging. It must be underlined that, in this study, the post-processing in the condylar region was limited to the smoothing process which allowed to “un-voxelize” the segmentation results, making the shape of the rendered model closer to that of the condyle. However, the present methodology involved specific post-segmentation procedures (mandibular superimposition, definition of plane cuts, and final registration) that were necessary to standardize the volumetric assessment of the condyles and the surface-to-surface matching technique. Since we could not exclude a priori potential errors related to these procedures, we performed intra-observer and inter-observer recordings even if previous evidence had confirmed that semi-automatic segmentation is not significantly influenced by operator reliability [1,22]. Manual segmentation presents two contrasting methodological aspects. First, it allows the definition of areas with low bone density or with no well-defined boundaries thanks to the manual adjustments and the intrinsic anatomical knowledge of the operator. This is the reason why manual segmentation is considered the gold standard in the absence of real anatomic structure or laser scanning procedure [18,42]. Second, the boundaries subsequently traced slide-by-slide by the operator may become mismatched, causing an alteration of the surface rendering [20]. This second factor explains why this methodology is trustworthy only when performed by an expert operator and is confirmed by the mean differences found in this study between the two operators (Table 5, Figure 7).

Based on the present findings and previous evidence [1] it can be asserted that (1) manual segmentation is trustworthy only in expert hands, but it is also extremely time-consuming, (2) there is still not sufficient evidence validating the accurate definition of a condylar region with semi-automatic method, although the procedure is extremely reliable and more efficient compared to the manual approach. Thus, from a clinical perspective, if a fine definition of condylar boundaries is required, clinicians with not adequate technical skills in 3D imaging, should refer to companies specialized in 3D imaging technology in order to overcome the segmentation accuracy and time management issues [42,43,44,45,46,47,48,49,50].

Recently, the application of artificial intelligence (AI), through its deep learning paradigm, has shown very promising results in automated segmentation of anatomical structures from CT and CBCT. In particular, convolutional neural networks (CNNs) [47,48,49,50,51] have led to a series of breakthroughs in CBCT segmentation [48], especially when compared to previous methods employing general hand-crafted features, thanks to learning task-specific features directly from data. Artificial intelligence for craniomaxillofacial structure bone segmentation can overcome two main limits of semi-automatic segmentation, i.e., the operator-dependent and time-consuming process and future studies are waited for this new open scenario.

## 5. Conclusions

Although semi-automatic segmentation is extremely reliable in 3D rendering models of the mandibular condylar region, according to the present findings, the range of error with this procedure approximates the clinical relevance. As a consequence, if an accurate definition of condylar boundaries is required, highly skilled clinicians can perform manual refinement. Otherwise, clinicians could refer to companies specialized in 3D imaging technology for this purpose.

## Figures and Tables

**Table 1:** Descriptive and general information of the software tested in the present study.

Name	Developer	Country	Operating System	Type	Segmentation Options
Mimics	Materialise	Belgium	Windows	Pay per use	Manual and semi-automatic
Invesalius	Information Tecnology Center Renato Archer	Brasil	Windows, Mac OS X, Linux	Open-source	Semi-automatic
Dolphin 3D	Patterson Dental Supply	USA	Windows	Pay per use	Semi-automatic
ITK-Snap	University of Pennsylvania and Utah	USA	Windows, Mac OS X, Linux	Open-source	Manual and semi-automatic
Slicer 3D	Harvard University	United Kingdom	Windows, Mac OS X, Linux	Open-source	Manual and semi-automatic

**Table 2:** Comparison among the volumetric measurements of the condylar 3D models obtained with the tested software. Significance set at *p* < 0.05, based on Kruskal–Wallis test; SE = standard error, NS = not significant.

	Sample	Median (mm^3^)	SE	Intervals		Significance
				Minimum	Maximum	
Mimics	20	1631.00	35.64	1402	1882	NS
Invesalius	20	1662.50	35.84	1416	1915	
Dolphin 3D	20	1650.00	35.29	1427	1885	
ITK-Snap	20	1602.00	34.56	1393	1864	
Slicer 3D	20	1614.50	36.26	1384	1875	

**Table 3:** Comparison among the matching percentage between the mandibular models obtained with different semi-automatic segmentation software and the ground truth mandibular model (manual segmentation), according to the deviation analysis. Significance set at *p* < 0.05, according to the Kruskal–Wallis test. Post-hoc comparisons performed with Mann–Whitney U test; SE = standard error; A = range of tolerance set at 0.3 mm; B = range of tolerance set at 0.6 mm.

		Sample	Median (%)	SE	Intervals		Significance
					Minimum	Maximum	
Tolerance A	Invesalius (a)	20	65,50 (b,c,d)	0,69	60,47	70,17	*p* < 0.001
	Dolphin 3D (b)	20	62,50 (a,c,d)	0,61	59,83	67,28	
	ITK-Snap (c)	20	69,00 (a,b,d)	0,89	64,32	74,08	
	Slicer 3D (d)	20	61,00 (a,b,c)	0,75	57,64	65,10	
Tolerance B	Invesalius (a)	20	80,20 (c)	0,55	77,71	84,18	*p* < 0.05
	Dolphin 3D (b)	20	78,50 (c)	0,91	74,91	84,1	
	ITK-Snap (c)	20	84,77 (a,b,d)	0,74	78,94	87,1	
	Slicer 3D (d)	20	82,55 (c)	0,66	76,92	84,83	

**Table 4:** Comparison between first and second volumetric readings of condylar models for each software (intra-observer reliability). Significance set at *p* < 0.05 according to the Wilcoxon Signed-Rank Test.

	Sample	First Reading Median (mm^3^)	Second Reading Median (mm^3^)	Significance
Mimics	20	1631	1624	*p* = 0.007
Invesalius	20	1663	1661	NS
Dolphin 3D	20	1650	1651	NS
ITK-Snap	20	1602	1612	NS
Slicer 3D	20	1615	1611	NS

**Table 5:** Comparison between first volumetric reading (operator 1) and second volumetric readings (operator 2) of condylar models for each software investigated (Inter-observer reliability). Significance set at *p* < 0.05 according to the Wilcoxon Signed-Rank Test.

	Sample	First Reading	Second Reading	Significance
		Median (mm^3^)	Median (mm^3^)	
Mimics	20	1631	1700	*p* < 0.001
Invesalius	20	1663	1668	NS
Dolphin 3D	20	1650	1670	NS
ITK-Snap	20	1602	1610	NS
Slicer 3D	20	1615	1625	NS

**Table 6:** Intra-operator and inter-operator reliability according to the intraclass correlation coefficient (ICC).

	Mimics	ITK-Snap	Invesalius	Dolphin 3D	Slicer 3D
	ICC	ICC	ICC	ICC	ICC
Intra-operator	0.933	0.996	0.996	0.997	0.997
Inter-operator	0.856	0.998	0.997	0.996	0.997

## References

[1] Kim J.J., Nam H., Kaipatur N.R., Major P.W., Flores-Mir C., Lagravere M.O., Romanyk D.L. (2019). Reliability and accuracy of segmentation of mandibular condyles from different three-dimensional imaging modalities: A systematic review. Dentomaxillofac. Radiol..

[2] Tecco S., Saccucci M., Nucera R., Polimeni A., Pagnoni M., Cordasco G., Festa F., Iannetti G. (2010). Condylar volume and surface in caucasian young adult subjects. BMC Med. Imaging.

[3] Nah K.S. (2012). Condylar bony changes in patients with temporomandibular disorders: A cbct study. Imaging Sci. Dent..

[4] Arnett G.W., Milam S.B., Gottesman L. (1996). Progressive mandibular retrusion-idiopathic condylar resorption. Part ii. Am. J. Orthod. Dentofac. Orthop..

[5] Hoppenreijs T.J., Freihofer H.P., Stoelinga P.J., Tuinzing D.B., van′t Hof M.A. (1998). Condylar remodelling and resorption after le fort i and bimaxillary osteotomies in patients with anterior open bite. A clinical and radiological study. Int. J. Oral Maxillofac. Surg..

[6] Nucera R., Lo Giudice A., Rustico L., Matarese G., Papadopoulos M.A., Cordasco G. (2016). Effectiveness of orthodontic treatment with functional appliances on maxillary growth in the short term: A systematic review and meta-analysis. Am. J. Orthod. Dentofac. Orthop..

[7] Nucera R., Militi A., Lo Giudice A., Longo V., Fastuca R., Caprioglio A., Cordasco G., Papadopoulos M.A. (2018). Skeletal and dental effectiveness of treatment of class ii malocclusion with headgear: A systematic review and meta-analysis. J. Evid. Based Dent. Pract..

[8] Zymperdikas V.F., Koretsi V., Papageorgiou S.N., Papadopoulos M.A. (2016). Treatment effects of fixed functional appliances in patients with class ii malocclusion: A systematic review and meta-analysis. Eur. J. Orthod..

[9] Xia J.J., Gateno J., Teichgraeber J.F. (2009). New clinical protocol to evaluate craniomaxillofacial deformity and plan surgical correction. J. Oral Maxillofac. Surg..

[10] Leonardi R., Muraglie S., Lo Giudice A., Aboulazm K.S., Nucera R. (2020). Evaluation of mandibular symmetry and morphology in adult patients with unilateral posterior crossbite: A cbct study using a surface-to-surface matching technique. Eur. J. Orthod..

[11] Alkhader M., Ohbayashi N., Tetsumura A., Nakamura S., Okochi K., Momin M.A., Kurabayashi T. (2010). Diagnostic performance of magnetic resonance imaging for detecting osseous abnormalities of the temporomandibular joint and its correlation with cone beam computed tomography. Dentomaxillofac. Radiol..

[12] Scarfe W.C., Farman A.G., Sukovic P. (2006). Clinical applications of cone-beam computed tomography in dental practice. J. Can. Dent. Assoc..

[13] Guerrero M.E., Jacobs R., Loubele M., Schutyser F., Suetens P., van Steenberghe D. (2006). State-of-the-art on cone beam ct imaging for preoperative planning of implant placement. Clin. Oral Investig..

[14] Cevidanes L.H., Hajati A.K., Paniagua B., Lim P.F., Walker D.G., Palconet G., Nackley A.G., Styner M., Ludlow J.B., Zhu H. (2010). Quantification of condylar resorption in temporomandibular joint osteoarthritis. Oral Surg. Oral Med. Oral Pathol. Oral Radiol. Endod..

[15] Xi T., van Loon B., Fudalej P., Berge S., Swennen G., Maal T. (2013). Validation of a novel semi-automated method for three-dimensional surface rendering of condyles using cone beam computed tomography data. Int. J. Oral Maxillofac. Surg..

[16] Bayram M., Kayipmaz S., Sezgin O.S., Küçük M. (2012). Volumetric analysis of the mandibular condyle using cone beam computed tomography. Eur. J. Radiol..

[17] da Silva R.J., Valadares Souza C.V., Souza G.A., Ambrosano G.M.B., Freitas D.Q., Sant′Ana E., de Oliveira-Santos C. (2018). Changes in condylar volume and joint spaces after orthognathic surgery. Int. J. Oral Maxillofac. Surg..

[18] Engelbrecht W.P., Fourie Z., Damstra J., Gerrits P.O., Ren Y. (2013). The influence of the segmentation process on 3d measurements from cone beam computed tomography-derived surface models. Clin. Oral Investig..

[19] Fourie Z., Damstra J., Schepers R.H., Gerrits P.O., Ren Y. (2012). Segmentation process significantly influences the accuracy of 3d surface models derived from cone beam computed tomography. Eur. J. Radiol..

[20] Xi T., Schreurs R., Heerink W.J., Bergé S.J., Maal T.J. (2014). A novel region-growing based semi-automatic segmentation protocol for three-dimensional condylar reconstruction using cone beam computed tomography (cbct). PLoS ONE.

[21] Dong T., Xia L., Cai C., Yuan L., Ye N., Fang B. (2019). Accuracy of in vitro mandibular volumetric measurements from cbct of different voxel sizes with different segmentation threshold settings. BMC Oral Health.

[22] El H., Palomo J.M. (2010). Measuring the airway in 3 dimensions: A reliability and accuracy study. Am. J. Orthod. Dentofac. Orthop..

[23] Leonardi R., Lo Giudice A., Rugeri M., Muraglie S., Cordasco G., Barbato E. (2018). Three-dimensional evaluation on digital casts of maxillary palatal size and morphology in patients with functional posterior crossbite. Eur. J. Orthod..

[24] Lo Giudice A., Ortensi L., Farronato M., Lucchese A., Lo Castro E., Isola G. (2020). The step further smile virtual planning: Milled versus prototyped mock-ups for the evaluation of the designed smile characteristics. BMC Oral Health.

[25] Lo Giudice A., Ronsivalle V., Grippaudo C., Lucchese A., Muraglie S., Lagravère M.O., Isola G. (2020). One Step before 3D Printing-Evaluation of Imaging Software Accuracy for 3-Dimensional Analysis of the Mandible: A Comparative Study Using a Surface-to-Surface Matching Technique. Materials (Basel).

[26] Leonardi R., Muraglie S., Bennici O., Cavallini C., Spampinato C. (2019). Three-dimensional analysis of mandibular functional units in adult patients with unilateral posterior crossbite: A cone beam study with the use of mirroring and surface-to-surface matching techniques. Angle Orthod..

[27] Katsumata A., Hirukawa A., Okumura S., Naitoh M., Fujishita M., Ariji E., Langlais R.P. (2007). Effects of image artifacts on gray-value density in limited-volume cone-beam computerized tomography. Oral Surg. Oral Med. Oral Pathol. Oral Radiol. Endod..

[28] Nicolielo L.F.P., Van Dessel J., Shaheen E., Letelier C., Codari M., Politis C., Lambrichts I., Jacobs R. (2017). Validation of a novel imaging approach using multi-slice ct and cone-beam ct to follow-up on condylar remodeling after bimaxillary surgery. Int. J. Oral Sci..

[29] Lo Giudice A., Rustico L., Caprioglio A., Migliorati M., Nucera R. (2020). Evaluation of condylar cortical bone thickness in patient groups with different vertical facial dimensions using cone-beam computed tomography. Odontology.

[30] Loreto C., Filetti V., Almeida L.E., La Rosa G.R.M., Leonardi R., Grippaudo C., Lo Giudice A. (2020). MMP-7 and MMP-9 are overexpressed in the synovial tissue from severe temporomandibular joint dysfunction. Eur. J. Histochem..

[31] Rosa M., Quinzi V., Marzo G. (2019). Paediatric Orthodontics Part 1: Anterior open bite in the mixed dentition. Eur. J. Paediatr. Dent..

[32] Paniagua B., Cevidanes L., Zhu H., Styner M. (2011). Outcome quantification using spharm-pdm toolbox in orthognathic surgery. Int. J. Comput. Assist. Radiol. Surg..

[33] Hatamleh M.M., Yeung E., Osher J., Huppa C. (2017). Novel treatment planning of hemimandibular hyperplasia by the use of three-dimensional computer-aided-design and computer-aided-manufacturing technologies. J. Craniofac. Surg..

[34] Ferro R., Besostri A., Olivieri A., Quinzi V., Scibetta D. (2016). Prevalence of cross-bite in a sample of Italian preschoolers. Eur. J. Paediatr. Dent..

[35] Isola G., Polizzi A., Iorio-Siciliano V., Alibrandi A., Ramaglia L., Leonardi R. (2020). Effectiveness of a nutraceutical agent in the non-surgical periodontal therapy: A randomized, controlled clinical trial. Clin. Oral Investig..

[36] Isola G., Alibrandi A., Currò M., Matarese M., Ricca S., Matarese G., Ientile R., Kocher T. (2020). Evaluation of salivary and serum ADMA levels in patients with periodontal and cardiovascular disease as subclinical marker of cardiovascular risk. J. Periodontol..

[37] Lo Giudice A., Caccianiga G., Crimi S., Cavallini C., Leonardi R. (2018). Frequency and type of ponticulus posticus in a longitudinal sample of nonorthodontically treated patients: Relationship with gender, age, skeletal maturity, and skeletal malocclusion. Oral Surg. Oral Med. Oral Pathol. Oral Radiol..

[38] Lo Giudice A., Brewer I., Leonardi R., Roberts N., Bagnato G. (2018). Pain threshold and temporomandibular function in systemic sclerosis: Comparison with psoriatic arthritis. Clin. Rheumatol..

[39] Leonardi R. (2019). Cone-beam computed tomography and three-dimensional orthodontics. Where we are and future perspectives. J. Orthod..

[40] Fischer B., Masucci C., Ruellas A., Cevidanes L., Giuntini V., Nieri M., Nardi C., Franchi L., McNamara J.A., Defraia E. (2018). Three-dimensional evaluation of the maxillary effects of two orthopaedic protocols for the treatment of class iii malocclusion: A prospective study. Orthod Craniofac. Res..

[41] Liang X., Lambrichts I., Sun Y., Denis K., Hassan B., Li L., Pauwels R., Jacobs R. (2010). A comparative evaluation of cone beam computed tomography (cbct) and multi-slice ct (msct). Part ii: On 3d model accuracy. Eur. J. Radiol..

[42] Lo Giudice A., Fastuca R., Portelli M., Militi A., Bellocchio M., Spinuzza P., Briguglio F., Caprioglio A., Nucera R. (2017). Effects of rapid vs slow maxillary expansion on nasal cavity dimensions in growing subjects: A methodological and reproducibility study. Eur. J. Paediatr. Dent..

[43] Spagnuolo G., Ametrano G., D’Antò V., Rengo C., Simeone M., Riccitiello F., Amato M. (2017). Effect of autoclaving on the surfaces of TiN -coated and conventional nickel-titanium rotary instruments. Int. Endod. J..

[44] Spagnuolo G., Ametrano G., D’Antò V., Formisano A., Simeone M., Riccitiello F., Amato M., Rengo S. (2012). Microcomputed tomography analysis of mesiobuccal orifices and major apical foramen in first maxillary molars. Open Dent. J..

[45] Ametrano G., D′Antò V., Di Caprio M.P., Simeone M., Rengo S., Spagnuolo G. (2011). Effects of sodium hypochlorite and ethylenediaminetetraacetic acid on rotary nickel-titanium instruments evaluated using atomic force microscopy. Int. Endod. J..

[46] D’Antò V., Eckhardt A., Hiller K.A., Spagnuolo G., Valletta R., Ambrosio L., Schmalz G., Schweikl H. (2009). The influence of Ni(II) on surface antigen expression in murine macrophages. Biomaterials.

[47] Isola G., Anastasi G.P., Matarese G., Williams R.C., Cutroneo G., Bracco P., Piancino M.G. (2018). Functional and molecular outcomes of the human masticatory muscles. Oral Dis..

[48] Isola G., Alibrandi A., Rapisarda E., Matarese G., Williams R.C., Leonardi R. (2020). Association of vitamin d in patients with periodontal and cardiovascular disease: A cross-sectional study. J. Periodontal. Res..

[49] Isola G., Polizzi A., Alibrandi A., Indelicato F., Ferlito S. (2020). Analysis of Endothelin-1 concentrations in individuals with periodontitis. Sci. Rep..

[50] Minnema J., van Eijnatten M., Kouw W., Diblen F., Mendrik A., Wolff J. (2018). Ct image segmentation of bone for medical additive manufacturing using a convolutional neural network. Comput. Biol. Med..

[51] Zhu Y., Wei R., Gao G., Ding L., Zhang X., Wang X., Zhang J. (2019). Fully automatic segmentation on prostate mr images based on cascaded fully convolution network. J. Magn. Reson. Imaging.

